# Prognostic and Immunomodulatory Roles of PAK6 in Colorectal Cancer Through Integrative Transcriptomic and Clinical Analysis

**DOI:** 10.3390/cancers17193183

**Published:** 2025-09-30

**Authors:** Chunxiang Ye, Guanjun Yue, Lei Yang, Zhenjun Wang

**Affiliations:** 1 Department of General Surgery, Beijing Chao-Yang Hospital, Capital Medical University, Beijing 100020, China; chunxiang-ye@163.com (C.Y.); yl6649084@mail.ccmu.edu.cn (L.Y.); 2 Department of Pathology, Peking University International Hospital, Beijing 102206, China; yueguanjun@pkuih.edu.cn

**Keywords:** PAK6, colorectal cancer, immune infiltration, prognosis, tumor microenvironment

## Abstract

**Simple Summary:**

Colorectal cancer remains a major health challenge worldwide, highlighting the need for new diagnostic and prognostic biomarkers. In this study, we investigated the role of PAK6, a protein kinase, in colorectal cancer through integrative analysis of transcriptomic and clinical data. We found that PAK6 is significantly upregulated in tumor tissues and is associated with aggressive disease features and poorer patient survival. Importantly, PAK6 expression correlates with immune cell infiltration and chemokine signaling, suggesting its involvement in shaping the tumor immune microenvironment. Our findings indicate that PAK6 emerges as a candidate biomarker worthy of further investigation in colorectal cancer, with potential implications for guiding immunotherapy strategies in the future.

**Abstract:**

Background: Colorectal cancer (CRC) represents a major global health challenge, characterized by rising incidence and mortality rates, necessitating improved diagnostic and therapeutic approaches. This study aimed to elucidate the expression and functional role of PAK6, a protein linked to cancer progression, as a potential biomarker for CRC. Methods: Utilizing comprehensive analyses of transcriptomic and clinical data from The Cancer Genome Atlas (TCGA) and Gene Expression Omnibus (GEO), we performed differential expression assessments, survival analyses, and functional enrichment studies. Results: Our findings demonstrate a significant upregulation of PAK6 in CRC tissues compared to adjacent normal tissues (*p* < 0.001), with a diagnostic AUC of 0.855, indicating its potential utility as a reliable biomarker for early detection. High PAK6 expression was significantly associated with aggressive clinicopathological features, including poor differentiation, residual tumor presence and reduced overall survival (HR = 1.72, *p* = 0.004). Functional enrichment analyses revealed PAK6’s involvement in critical biological processes such as cell cycle regulation, alongside its correlation with immune infiltration, particularly NK and CD8^+^ T cells. Moreover, PAK6 expression positively correlated with chemokines involved in immune cell recruitment, suggesting its role in modulating the tumor immune microenvironment. Conclusions: Our study underscores the significance of PAK6 as a diagnostic and prognostic biomarker in CRC, with the potential to inform targeted therapeutic strategies and enhance patient outcomes. Future research should focus on validating these findings in larger cohorts and exploring PAK6-targeted interventions to improve immunotherapeutic responses in CRC patients

## 1. Introduction

Colorectal cancer (CRC) stands as one of the most prevalent malignancies globally, imposing a substantial burden on public health [1,2]. Despite remarkable advancements in surgical techniques, chemotherapy, and targeted therapies, the prognosis for CRC patients, particularly those with advanced—stage disease, remains suboptimal [3,4]. As such, there is an urgent need to identify novel molecular biomarkers and therapeutic targets to enhance the diagnosis, treatment, and prognosis of CRC.

P21-activated kinase 6 (PAK6), a member of the PAK family of serine/threonine kinases, has been implicated in a variety of cellular processes, including cell proliferation, survival, migration, and invasion [5,6]. Previous studies have demonstrated that abnormal expression of PAK6 is associated with the development and progression of several types of cancer, such as breast cancer, cervical cancer and prostate cancer [7,8,9]. However, the role of PAK6 in CRC remains poorly understood.

In the context of CRC research, extensive efforts have been dedicated to elucidating the molecular mechanisms underlying tumorigenesis and progression [10,11]. Biomarkers such as KRAS, BRAF, and microsatellite instability have been identified and have significantly influenced treatment decisions [12,13]. Nevertheless, there are still gaps in our understanding of the complex molecular network in CRC. The function of PAK6 in CRC, including its expression pattern, clinical significance, and relationship with tumor immunity, has not been comprehensively investigated.

The tumor immune microenvironment has emerged as a critical determinant of cancer development and response to treatment [14,15]. Immune cells, such as CD8^+^ T cells, natural killer cells, and macrophages, play crucial roles in either promoting or inhibiting tumor growth [16,17,18,19]. Understanding the relationship between PAK6 and the tumor immune microenvironment in CRC could provide new insights into the disease mechanism and potential immunotherapeutic strategies.

In this study, we aimed to fill this knowledge gap by employing bioinformatics methods to analyze the expression, clinical significance, and tumor-immune relationship of the PAK6 gene in CRC. Additionally, we validated our findings through immunohistochemical detection of tissue microarrays. We hypothesized that PAK6 might play a crucial role in CRC development and progression and could serve as a potential biomarker and therapeutic target for this disease.

## 2. Materials and Methods

### 2.1. Data Acquisition and Preprocessing

Transcriptomic data and clinical information for pan-cancer analysis were retrieved from The Cancer Genome Atlas (TCGA) through the Xiantao academic online analysis platform (https://www.xiantaozi.com/, accessed on 23 February 2025). For colon cancer-specific analyses, RNA-seq data from 521 colon adenocarcinoma (COAD) samples (including 41 paired normal tissues, TCGA-COAD 521 cohort) were processed using the Toil workflow for uniform normalization and log2 transformation. Raw data from the GEO dataset GSE10950 (colon cancer), GSE41328 (colorectal cancer), GSE110224 (colorectal cancer) were downloaded, and the “limma” package of R software (Version 4.4.3) was used to identify DEGs across experimental condition, and the significant DEGs with *p* < 0.05 and [logFC] > 1.0 were considered as the cut-off criteria. The heatmap, UMAP, volcano plot and box plot were conducted by using “Complex Heatmap”, “umap” and “ggplot2” packages of R software (Version 4.4.3). In this study, the data from public databases was obtained between February and May 2025.

### 2.2. Expression Profiling and Diagnostic Evaluation

Differential expression of PAK6 across 33 tumor types was analyzed using the Wilcoxon rank-sum test. For paired COAD samples, the Wilcoxon signed-rank test was applied. Receiver operating characteristic (ROC) curves were generated using the “pROC” package (v1.18.0) with area under the curve (AUC) calculations to assess PAK6’s diagnostic potential. Tumor purity adjustments were performed using the ESTIMATE algorithm prior to analysis.

### 2.3. Clinical Correlation and Survival Analysis

Associations between PAK6 expression and clinicopathological parameters (TNM staging, histological type, etc.) were evaluated via *t*-test. Kruskal–Wallis tests for ordinal variables and Spearman correlation for continuous variables. Kaplan–Meier survival curves stratified by median PAK6 expression were generated using the “survival” package (v3.3-1), with log-rank tests for significance. Multivariate Cox proportional hazards models incorporated age, gender, stage, etc., with hazard ratios (HRs) reported using 95% confidence intervals.

### 2.4. Co-Expression Network and Functional Enrichment

Genes significantly correlated with PAK6 (|Pearson r| > 0.3, FDR-adjusted *p* < 0.05) were identified using TCGA-COAD data. The top 15 positively/negatively correlated genes were visualized in a heatmap via ggplot2 package. Gene Ontology (GO) and Kyoto Encyclopedia of Genes and Genomes (KEGG) enrichment analyses were performed using “clusterProfiler” (v4.0.5) with the top 100 co-expressed genes. Gene Set Enrichment Analysis (GSEA) utilized hallmark gene sets from MSigDB (v7.4) with 1000 permutations and FDR < 0.25 as significance thresholds.

### 2.5. Immune Microenvironment Characterization

Immune cell infiltration scores were calculated for GSE10950 using ESTIMATE (Estimation of Stromal and Immune cells in Malignant Tumours using Expression data) algorithm. The TIMER2.0 algorithm (http://timer.cistrome.org/, accessed on 23 February 2025) quantified immune cell abundances (NK cells, CD4^+^ T cells, CD8^+^ T cells, cancer associated fibroblast cells) in TCGA-COAD, with Spearman correlations to PAK6 expression. Immune checkpoint genes (CD276, LAG3, etc.) and immunomodulatory gene sets (ImmPort) were analyzed for overlap with PAK6-associated genes.

### 2.6. Chemokine Interaction Network

A comprehensive chemokine-related gene list was compiled from GO (GO:0008009), KEGG (hsa04062), Reactome (R-HSA-380108), and MSigDB (M5911). Intersection with PAK6-correlated genes (*p* < 0.01) identified putative chemokine regulators. The TISIDB portal (http://cis.hku.hk/TISIDB/, accessed on 9 May 2025) validated associations between PAK6 and chemokine ligand/receptor pairs using Spearman correlation.

### 2.7. Tissue Microarray and Immunohistochemistry

Formalin-fixed paraffin-embedded (FFPE) tissues from 49 CRC patients (2020–2023, Beijing Chaoyang Hospital) were made into tissue microarray (TMA). All sample donors provided informed consent for participation in this study, which was approved by the Institutional Ethics Committee of Beijing Chao-Yang Hospital, Capital Medical University (2018-ke-99). The PAK6 antibody (Cat No. 13539-1-AP, Proteintech Group, Inc., Wuhan, China) was used for immunohistochemical detection. The staining results were evaluated by two independent pathologists using the semi-quantitative H-score system. The median of the H-score was used as the cut-off value to divide them into the high-expression group and the low-expression group.

### 2.8. Statistical Analysis

All statistical analyses were conducted using R software (version 4.4.3) unless otherwise specified. Differential expression analysis between tumor and normal tissues was assessed using Wilcoxon rank-sum tests for unpaired samples and Wilcoxon signed-rank tests for paired specimens. Associations between PAK6 expression and clinicopathological parameters were evaluated through Welch’s one-way ANOVA with Bonferroni post hoc adjustments for continuous variables and Pearson’s chi-square tests for categorical variables. Fisher’s exact probability method was applied when chi-square assumptions (expected frequency > 5, sample size > 40) were violated.

Survival outcomes including overall survival (OS), disease-specific survival (DSS), and progression-free interval (PFI) were analyzed using Kaplan–Meier methodology with log-rank tests for significance comparison. Prognostic independence was determined through univariate and multivariate Cox proportional hazards regression analyses, incorporating all variables demonstrating *p* < 0.1 in preliminary univariate screening. Prior to multivariate Cox regression, variance inflation factors (VIFs) were calculated to assess multicollinearity. All variables had VIFs < 10, indicating no severe multicollinearity. The proportional hazards assumption for the final multivariate Cox model was tested globally and for each variable using Schoenfeld residuals. The global test was not significant (*p* > 0.05), supporting the overall validity of the model.

For large-scale analyses, including differential expression, immune cell correlation, and Gene Set Enrichment Analysis (GSEA), *p*-values were adjusted for the false discovery rate (FDR) using the Benjamini–Hochberg method, and adjusted q-values are reported. All correlation analyses were performed using Spearman’s rank correlation method. Diagnostic performance was evaluated via receiver operating characteristic (ROC) curve analysis using the pROC package (v1.18.0). Correlation analyses employed Spearman’s rank method with Bonferroni correction for multiple comparisons. All statistical tests were two-tailed, and *p* ≤ 0.05 was significant for all tests.

## 3. Results

### 3.1. Aberrant Upregulation of PAK6 mRNA and Protein in Colorectal Cancer

**Figure 1:**
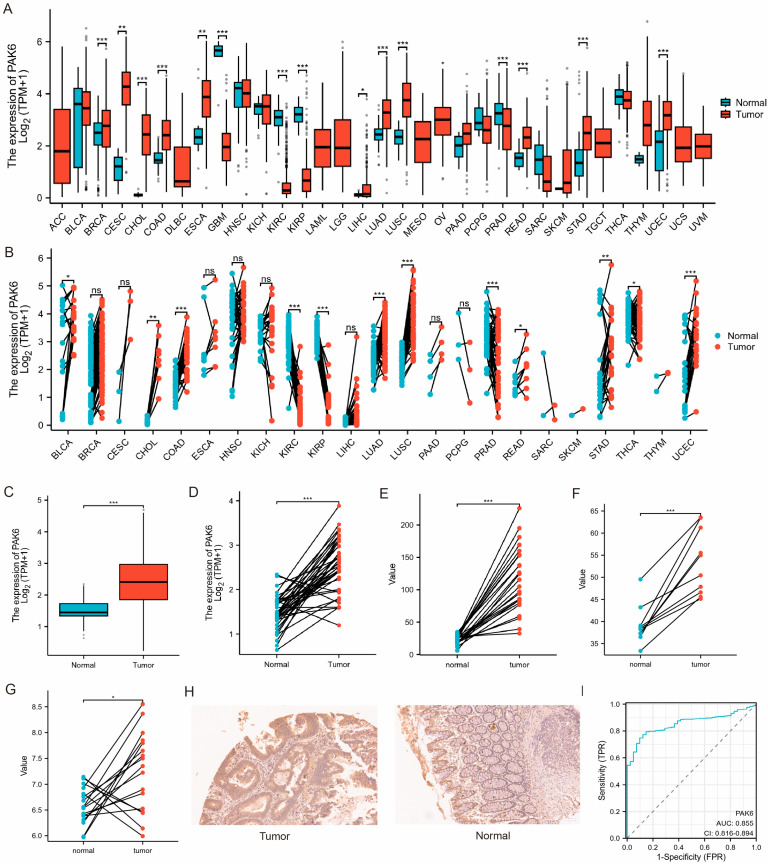
PAK6 is significantly overexpressed in colorectal cancer and other human malignancies. (**A**,**B**) PAK6 mRNA expression is upregulated in multiple cancer types compared to adjacent normal tissues based on TCGA pan-cancer analysis. CESC, cervical squamous cell carcinoma; CHOL, cholangiocarcinoma; ESCA, esophageal carcinoma; LUAD, lung adenocarcinoma; STAD, stomach adenocarcinoma; UCEC, uterine corpus endometrial carcinoma. (**C**,**D**) PAK6 mRNA expression is significantly elevated in colorectal cancer (CRC) tumor tissues compared to normal colon samples from TCGA database (*p* < 0.001). (**E**–**G**) Validation of PAK6 upregulation in CRC using three independent GEO datasets (GSE10950, GSE41328, GSE110224) in paired tumor-normal samples. (**H**) Immunohistochemical (IHC) staining of PAK6 in a colon cancer tissue microarray confirms higher protein expression in tumor tissues compared to paired normal tissues (*p* < 0.001). (**I**) Receiver operating characteristic (ROC) curve analysis of PAK6 expression for distinguishing CRC from normal tissues, with an AUC of 0.855 (95% CI: 0.812–0.898). Data are presented as mean ± SEM; statistical significance was determined by Student’s *t*-test or ANOVA; ns, not significant; * *p* < 0.05, ** *p* < 0.01, *** *p* < 0.001.

Pan-cancer analysis of TCGA data revealed significant overexpression of PAK6 mRNA in tumor tissues compared to adjacent normal tissues across multiple malignancies, including cervical squamous cell carcinoma (CESC), cholangiocarcinoma (CHOL), esophageal carcinoma (ESCA), lung adenocarcinoma (LUAD), stomach adenocarcinoma (STAD), and uterine corpus endometrial carcinoma (UCEC) (Figure 1A,B). In colon cancer, PAK6 expression was markedly elevated in tumor tissues versus normal colon samples (Figure 1C,D, *p* < 0.001). Validation across three independent GEO datasets (GSE10950, GSE41328, GSE110224) demonstrated consistent findings in paired CRC tumor-normal cohorts (Figure 1E–G). Subsequently, we detected the expression of PAK6 in colon cancer tumor tissues and paired normal tissues in the tissue microarray by immunohistochemistry. The results showed that the expression of PAK6 in tumor tissues was higher than that in normal tissues (Figure 1H, *p* < 0.001). ROC analysis confirmed PAK6’s diagnostic utility, yielding an AUC of 0.855 (95 CI: 0.812–0.898) for distinguishing CRC from normal tissue (Figure 1I).

### 3.2. Association of PAK6 with Aggressive Clinicopathological Features

**Figure 2:**
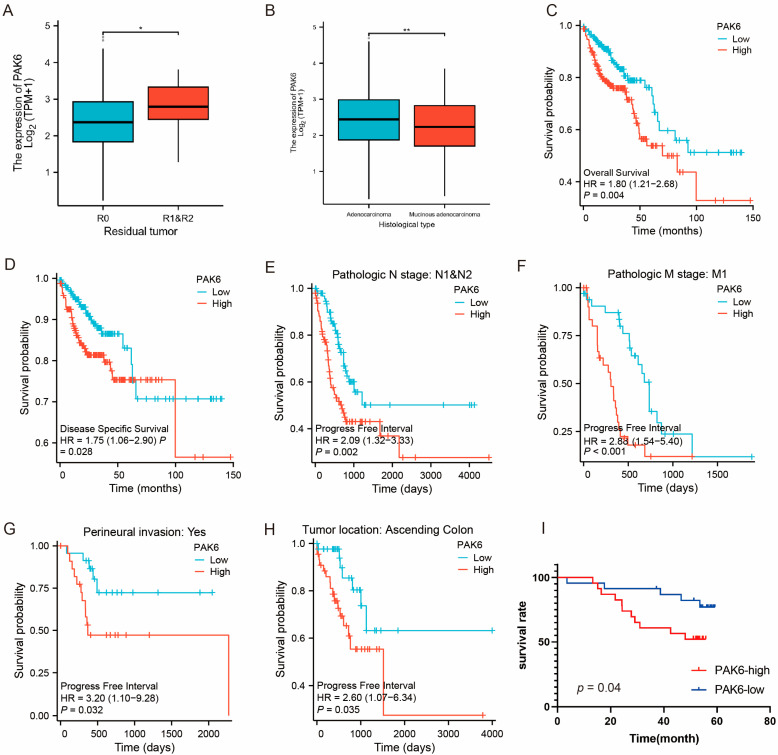
High PAK6 expression is associated with aggressive clinicopathological features and poor prognosis in colorectal cancer. (**A**,**B**) Analysis of TCGA-CRC cases reveals that high PAK6 expression is significantly associated with the presence of residual tumor (**A**, *p* = 0.02) and specific histological subtypes (**B**, *p* = 0.005). (**C**,**D**) Kaplan–Meier survival analysis shows that patients with high PAK6 expression have significantly worse overall survival (OS, **C**; HR = 1.72, *p* = 0.004) and disease-specific survival (DSS, **D**; HR = 1.54, *p* = 0.028) compared to those with low PAK6 expression. (**E**–**H**) Subgroup analyses of progression-free interval (PFI) demonstrate that high PAK6 expression is associated with worse outcomes in specific patient cohorts: those with lymph node metastasis (N1/N2, **E**; *p* = 0.002), distant metastasis (M1, **F**; *p* < 0.001), peritoneal metastasis (**G**; *p* = 0.032), right-sided colon cancer (**H**; *p* = 0.035). (**I**) Validation by immunohistochemistry (IHC) on tissue microarrays confirms that high PAK6 protein expression is associated with significantly poorer overall survival in an independent CRC cohort (*p* = 0.04). Statistical significance was determined by Chi-square test (**A**,**B**), Log-rank test (**C**–**I**); HR, hazard ratio; * *p* < 0.05; ** *p* < 0.01.

Analysis of 521 TCGA-COAD cases revealed significant correlations between high PAK6 expression and residual tumor presence (Figure 2A, *p* = 0.02) and histological subtypes (Figure 2B, *p* = 0.005). While no associations were observed with T/N/M staging or overall TNM stage, survival analyses demonstrated prognostic significance. Patients with PAK6-high tumors exhibited reduced overall survival (OS: HR = 1.72, *p* = 0.004) and disease-specific survival (DSS: HR = 1.54, *p* = 0.028) compared to PAK6-low counterparts (Figure 2C,D). Subgroup analyses identified significant progression-free interval (PFI) correlations in lymph node-positive (Figure 2E, *p* = 0.002), metastatic (Figure 2F, M1, *p* < 0.001), peritoneal metastasis (Figure 2G, *p* = 0.032), right-sided colon cancer (Figure 2H, *p* = 0.035), and R2-resected (*p* = 0.037) cohorts.

Univariate Cox regression identified advanced T-stage (T3/T4, *p* = 0.004), nodal involvement (N1/N2, *p* < 0.001), metastasis (M1, *p* < 0.001), advanced TNM stage (III/IV, *p* < 0.001), PAK6-high status (*p* = 0.004), poor primary therapy response (PD/SD, *p* < 0.001), age > 65 years (*p* = 0.028), incomplete resection (R1/R2, *p* < 0.001), and lymphatic invasion (*p* < 0.001) as adverse prognostic factors for OS (Table 1). The proportional hazards assumption was satisfied for the multivariate model (global test, *p* > 0.05). Multivariate analysis identified nodal involvement (N1/N2, HR = 2.11, *p* < 0.001), poor response to primary therapy (PD/SD, HR = 3.02, *p* < 0.001), and the presence of lymphatic invasion (HR = 1.89, *p* = 0.01) as independent predictors of poorer overall survival (Table 1).

We next analyzed the results of immunohistochemistry on tissue microarrays, which revealed that the expression level of PAK6 was significantly associated with tumor differentiation (*p* = 0.021), but not with any other clinicopathological factors (Table 2). Survival analysis revealed that PAK6 expression (*p* = 0.04, Figure 2I), differentiation degree (*p* = 0.002), perineural invasion (*p* = 0.032), lymph node metastasis (*p* = 0.027), distant metastasis (*p* = 0.012), and tumor stage (*p* = 0.002) were associated with the prognosis of CRC patients. However, in the multivariate COX regression analysis, only tumor stage (*p* = 0.006) emerged as an independent prognostic factor for CRC patients.

### 3.3. Functional Enrichment Implicates PAK6 May Be Involved in Malignant Progression of CRC

**Figure 3:**
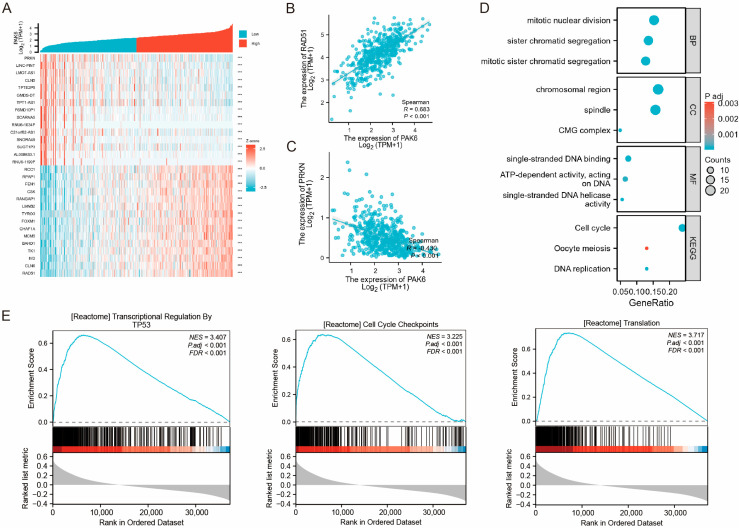
Functional enrichment analysis reveals PAK6 is involved in cell cycle regulation and malignant progression of CRC. (**A**) Heatmap of the top 15 genes most significantly positively and negatively correlated with PAK6 expression in the TCGA-CRC cohort. (**B**) Scatter plots showing the strongest positive correlation between PAK6 and RAD51 (Pearson’s R = 0.683, *p* < 0.001) and (**C**) the strongest negative correlation between PAK6 and PRKN (Pearson’s R = −0.430, *p* < 0.001). (**D**) Bar plot of significantly enriched Gene Ontology (GO) terms and Kyoto Encyclopedia of Genes and Genomes (KEGG) pathways for genes co-expressed with PAK6. Biological Processes (BP), Cellular Components (CC), and Molecular Functions (MF) are shown. The most enriched terms and pathways are related to chromosome segregation, nuclear division, and DNA replication. (**E**) Gene Set Enrichment Analysis (GSEA) plots showing that genes correlated with high PAK6 expression are significantly enriched in gene sets related to transcriptional regulation by TP53, cell cycle checkpoints, and translation. The *p* values in (**D**,**E**) are both adjusted by FDR. (*** *p* < 0.001).

To elucidate the biological functions of PAK6 in colorectal cancer (CRC) pathogenesis, we systematically analyzed genes exhibiting significant correlations with PAK6 expression. From the TCGA database, we identified the top 100 genes showing the strongest positive and negative correlations with PAK6 expression, respectively. Figure 3A displays a heatmap illustrating the 15 most significantly positively and negatively correlated genes. Notably, RAD51 demonstrated the strongest positive correlation (Pearson’s R = 0.683, *p* < 0.001; Figure 3B), while PRKN exhibited the most pronounced negative correlation (Pearson’s R = −0.430, *p* < 0.001; Figure 3C).

Collectively, these results implicate PAK6 in the malignant progression of CRC by potentially modulating critical processes such as cell cycle dynamics, DNA replication, and mitotic regulation.

Subsequent GO/KEGG enrichment analyses revealed distinct functional patterns. For GO terms, the top three enriched biological processes (BP) were sister chromatid segregation, mitotic sister chromatid segregation, and mitotic nuclear division. Cellular component (CC) analysis highlighted enrichment in chromosomal region, spindle, and CMG complex. Molecular functions (MF) predominantly involved single-stranded DNA helicase activity, single-stranded DNA binding, and ATP-dependent activity (acting on DNA). KEGG pathway analysis identified Cell cycle, DNA replication, and Oocyte meiosis as the most significantly enriched pathways associated with PAK6 (Figure 3D).

Gene Set Enrichment Analysis (GSEA) further demonstrated that PAK6-correlated genes were primarily enriched in transcriptional regulation by TP53, cell cycle checkpoints, and translation pathways (Figure 3E).

Collectively, these findings suggest that PAK6 may critically contribute to malignant phenotypic progression and tumorigenesis in CRC by modulating cell cycle dynamics and mitotic regulation.

### 3.4. PAK6 Expression Negatively Correlates with Tumor Microenvironment Scores and Influences Immune Cell Infiltration

**Figure 4:**
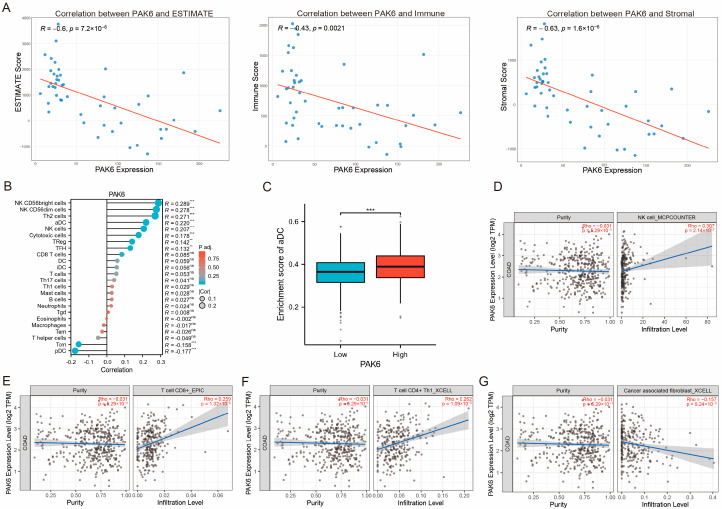
PAK6 expression is negatively correlated with tumor microenvironment scores and influences immune cell infiltration in colorectal cancer. (**A**) Scatter plots showing significant negative correlations between PAK6 expression and tumor microenvironment scores (ESTIMATE, Immune, and Stromal scores) derived from the ESTIMATE algorithm in the GSE10950 dataset. (**B**) Lollipop plot illustrating the Spearman correlation coefficients between PAK6 expression and infiltration levels of various immune cell subsets, as assessed by single-sample Gene Set Enrichment Analysis (ssGSEA). NK, natural killer; aDC, activated dendritic cell; pDC, plasmacytoid dendritic cell; Tcm, central memory T cell; TFH, follicular helper T cell. (**C**) Comparative analysis of activated dendritic cell (aDC) enrichment between PAK6-high and PAK6-low expression groups in the TCGA cohort (*p* < 0.001). (**D**–**G**) Correlation analysis using TIMER2.0 reveals that PAK6 expression is positively associated with the infiltration levels of NK cells (**D**), CD8^+^ T cells (**E**), and CD4^+^ T cells (**F**), and negatively associated with cancer-associated fibroblasts (CAFs) (**G**) in the TCGA-CRC cohort. Data are presented as mean ± SEM; statistical significance was determined by Pearson or Spearman correlation analysis (**A**,**D**–**G**), and Student’s *t*-test (**C**). *p*-values were adjusted for the FDR for (**B**). (ns, not significant; ** *p* < 0.01; *** *p* < 0.001).

To investigate the relationship between PAK6 expression and immune cell infiltration in colorectal cancer tissues, we conducted comprehensive analyses using multiple computational approaches. First, the ESTIMATE algorithm applied to the GSE10950 dataset revealed significant negative correlations between PAK6 expression and tumor microenvironment scores: ESTIMATE score (R = −0.6, *p* = 7.2 × 10^−6^), Immune score (R = −0.43, *p* = 0.0021), and Stromal score (R = −0.63, *p* = 1.6 × 10^−6^) (Figure 4A).

Second, single-sample Gene Set Enrichment Analysis (ssGSEA), which was performed in the TCGA-COAD cohort of 521 samples, demonstrated Spearman correlations between PAK6 expression and infiltration levels of specific immune subsets, including NK CD56 bright cells, NK CD56dim cells, Th2 cells, activated dendritic cells (aDC), cytotoxic cells, plasmacytoid dendritic cells (pDC), central memory T cells (Tcm), and follicular helper T cells (TFH) (Figure 4B and Table 3).

Third, comparative analysis in the TCGA-COAD cohort of 521 samples showed significantly higher enrichment of activated dendritic cells in PAK6-high versus PAK6-low expression groups (*p* < 0.001, Figure 4C).

Finally, TIMER2.0-based correlation analysis further revealed that PAK6 expression positively associated with NK cells (R = 0.28, *p* < 0.001, Figure 4D), CD8^+^ T cells (R = 0.19, *p* = 0.003, Figure 4E), and CD4^+^ T cells (R = 0.23, *p* = 0.001, Figure 4F), while negatively correlating with cancer-associated fibroblasts (R = −0.35, *p* = 2.1 × 10^−5^, Figure 4G).

Collectively, these findings suggest that PAK6 may modulate tumor immunity by reshaping immune cell composition within the tumor microenvironment.

### 3.5. PAK6 Is Linked to Immune Regulatory Genes and Checkpoint Molecules

**Figure 5:**
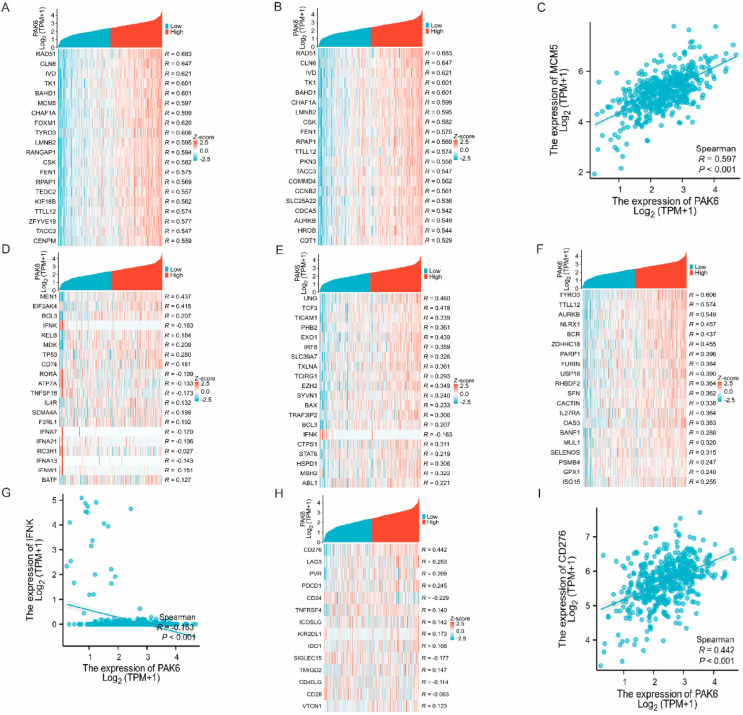
PAK6 expression is associated with immune regulatory genes and checkpoint molecules in colorectal cancer. (**A**,**B**) Heatmaps displaying the correlation between PAK6 expression and a panel of immunostimulatory (**A**) and immunosuppressive (**B**) genes in the CRC tumor microenvironment from the TCGA cohort. (**C**) Scatter plot validating the strongest positive correlation between PAK6 and MCM5 (R = 0.597, *p* < 0.001), an immunostimulatory gene. (**D**–**F**) Heatmaps showing significant enrichment of PAK6-correlated genes in immune-related biological processes, including T cell activation (**D**), B cell activation (**E**), and negative regulation of immune response (**F**). (**G**) Scatter plot showing a significant negative correlation between PAK6 and IFNK (R = −0.163, *p* < 0.001), a key gene involved in T and B cell activation pathways. (**H**,**I**) Analysis of immune checkpoint molecules reveals significant positive correlations between PAK6 expression and key checkpoint proteins CD276 (R = 0.44, *p* < 0.001), LAG3 (R = 0.30, *p* < 0.001), and PVR (R = 0.27, *p* < 0.001), as visualized in the heatmap (**H**) and scatter plots (**I**).

To delineate the immunomodulatory role of PAK6 in colorectal cancer, we systematically evaluated its correlations with immune regulatory genes in the TCGA-COAD 521 cohort. First, PAK6 exhibited significant associations with numerous immunostimulatory and immunosuppressive genes within the CRC tumor microenvironment (Figure 5A,B). Among these, several genes play dual regulatory roles in immune modulation, exerting both immunosuppressive and immunostimulatory effects, such as RAD51, CLN6 and IVD. MCM5 has been confirmed as an oncogene in colon adenocarcinoma, promoting tumor progression by regulating the cell cycle. As an immunostimulatory gene, its expression in colon cancer shows a significant positive correlation with PAK6 (Figure 5C).

Second, via the ImmPort database, we analyzed PAK6’s associations with immune-related gene sets, revealing its close linkage to T cell activation, B cell activation, and negative regulation of immune response (Figure 5D–F). A critical observation was the significant negative correlation between PAK6 and IFNK (R = −0.163, *p* < 0.001), a dual-function gene involved in both T and B cell activation pathways (Figure 5G).

Finally, immune checkpoint analysis identified significant correlations between PAK6 expression and key checkpoint molecules: CD276 (R = 0.44, *p* < 0.001), LAG3 (R = 0.30, *p* < 0.001), and PVR (R = 0.27, *p* < 0.001) (Figure 5H,I). These findings suggest PAK6 may regulate immune checkpoint dynamics in the tumor microenvironment, potentially influencing responses to immune checkpoint inhibitors.

### 3.6. PAK6 Is Associated with Chemokine Signaling and Immune Cell Migration

**Figure 6:**
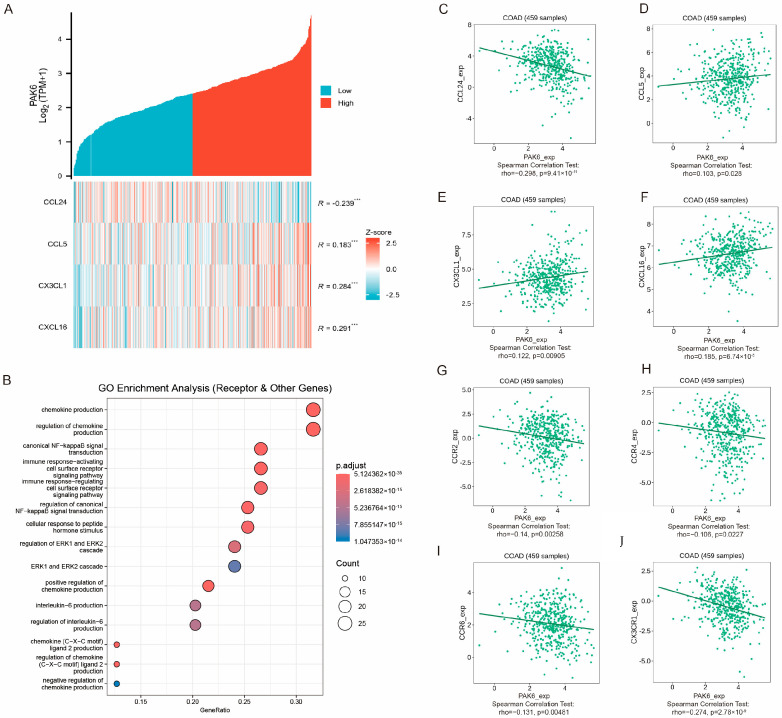
PAK6 expression is correlated with chemokine signaling and may regulate immune cell migration in colorectal cancer. (**A**) Heatmap demonstrating significant positive correlations (all *p* < 0.001) between PAK6 expression and key chemokines (CCL24, CCL5, CX3CL1, CXCL16) in the TCGA-CRC cohort, with CXCL16 showing the strongest correlation (R = 0.291). (**B**) Bubble plot of Gene Ontology (GO) enrichment analysis showing that PAK6-associated chemokine receptors and pathway genes are predominantly enriched in biological processes related to chemokine production and regulation. (**C**–**J**) Independent validation using the TISIDB database confirms significant correlations (*p* < 0.05) between PAK6 expression and the four highlighted chemokines (**C**–**F**) as well as key chemokine receptors CCR2, CCR4, CCR6, and CX3CR1 (**G**–**J**) in CRC. (*** *p* < 0.001).

To further investigate the role of PAK6 in immune cell migration, activation, and response, we analyzed its associations with chemokines and chemokine receptors in the TCGA-COAD 521 cohort. By integrating gene lists from multiple databases (GO, KEGG, MSigDB, Reactome), we identified significant positive correlations between PAK6 expression and chemokines including CCL24, CCL5, CX3CL1, and CXCL16 (all *p* < 0.001), with CXCL16 showing the strongest correlation (R = 0.291, Figure 6A). Subsequent clustering-based enrichment analysis of PAK6-associated chemokine receptors and pathway genes revealed predominant enrichment in GO terms related to chemokine production and regulation of chemokine production (Figure 6B). Finally, we validated the association between PAK6 and the identified chemokine network in colorectal cancer using the TISIDB database. This independent verification confirmed significant correlations (*p* < 0.05) between PAK6 expression and the four previously highlighted chemokines (CCL24, CCL5, CX3CL1, CXCL16), along with key chemokine receptors including CCR2, CCR4, CCR6, and CX3CR1 (Figure 6C–J). These findings collectively suggest that PAK6 might participate in regulating the directional migration and recruitment of immune cells.

## 4. Discussion

In this comprehensive study, we integrated bioinformatics analysis and immunohistochemical validation to explore the role of PAK6 in CRC. Our bioinformatics results, derived from multiple datasets, consistently demonstrated that PAK6 was significantly upregulated in CRC tissues compared with normal tissues. This finding was further corroborated by our immunohistochemical analysis of tissue microarrays. Previous studies on other cancer types, such as breast cancer [20], lung cancer [21], prostate cancer [22], and cervical cancer [9], have also reported elevated PAK6 expression, which is in line with our results in CRC. The upregulation of PAK6 in CRC may be attributed to genetic alterations, such as gene amplification or promoter hypomethylation, although further investigations are required to clarify the exact molecular mechanisms.

We also found a significant association between PAK6 expression and clinicopathological features of CRC patients. High PAK6 expression was closely related to advanced tumor stage, poor differentiation, and lymph node metastasis. These results suggest that PAK6 may contribute to CRC progression by promoting tumor cell proliferation, invasion, and metastasis. Similar associations between PAK6 expression and aggressive clinicopathological features have been observed in other malignancies [23,24,25], indicating a conserved role of PAK6 in cancer progression.

In terms of tumor immunity, our bioinformatics analysis revealed a remarkable correlation between PAK6 expression and CRC immune cell infiltration: high PAK6 was associated with increased CD8^+^ T cells, natural killer cells, and macrophages, implying its role in modulating the tumor immune microenvironment—critical for cancer development and treatment response t [14,15,26]. However, PAK6 also negatively correlated with immune scores, suggesting elevated PAK6 may foster immunosuppression, potentially impairing immunotherapy efficacy [27]. Our data suggest a model where high PAK6 expression is associated with an ‘infiltrated but dysfunctional’ tumor immune landscape. The concurrent increase in cytotoxic immune cells and upregulation of immunosuppressive checkpoints (e.g., CD276, LAG3) points towards an environment where anti-tumor immunity may be suppressed. This is similar to the regulatory effects of some other genes on the tumor immune microenvironment, for instance, signal transducer and activator of transcription 3 (STAT3) plays a dual role in tumor immunity by enhancing immune cell recruitment to tumors while also promoting an immunosuppressive environment that hinders T and NK cell function [28,29]. Additionally, PAK6 correlated positively with key chemokines, indicating it might have a potential role in modulating immune cell recruitment/trafficking via the chemokine network. Clarifying PAK6’s exact immune role in CRC is needed, but these findings highlight its multifaceted impact on the immune landscape, supporting it as a target for therapies combining PAK6 inhibition and chemokine modulation to enhance anti-tumor immunity [27,30].

However, our study has several limitations. First, although our bioinformatics analysis was based on multiple publicly available datasets, potential batch effects and differences in data processing methods among these datasets may have influenced the results. Second, our immunohistochemical validation was limited to a relatively small number of tissue samples, which may not fully represent the entire CRC patient population. Third, it is important to note that the associations identified here are observational and do not establish causality. Therefore, our findings should be interpreted as generating a strong hypothesis that warrants future validation.

Despite these limitations, our study provides valuable insights into the role of PAK6 in CRC. The identification of PAK6 as a potential biomarker and therapeutic target in CRC may have important implications for the diagnosis, treatment, and prognosis of this disease. Future studies using in vitro and in vivo models are essential to elucidate the mechanistic role of PAK6 in CRC progression and to formally test its potential as a therapeutic target, as well as its role in tumor immunity. Additionally, larger-scaled, multi-centered, prospective clinical studies are needed to establish the clinical utility of PAK6 in CRC.

## 5. Conclusions

In conclusion, our study demonstrates that PAK6 is upregulated in CRC and is associated with clinicopathological features and tumor immunity. PAK6 may serve as a potential biomarker and therapeutic target for CRC. Further research in this area is warranted to fully understand the role of PAK6 in CRC and to develop more effective strategies for the treatment of this disease.

## Figures and Tables

**Table 1:** The univariate and multivariate analysis of Overall Survival (OS) *.

Characteristics	Total (N)	HR(95% CI) Univariate Analysis	*p* Value Univariate Analysis	HR (95% CI) Multivariate Analysis	*p* Value Multivariate Analysis
Pathologic T stage	476				
T1 & T2	94	Reference		Reference	
T3 & T4	382	3.072 (1.423–6.631)	0.004	279,063.4392 (0.000–Inf)	0.992
Pathologic N stage	477				
N0	283	Reference		Reference	
N1 & N2	194	2.592 (1.743–3.855)	<0.001	0.003 (0.000–0.078)	<0.001
Pathologic M stage	414				
M0	348	Reference		Reference	
M1	66	4.193 (2.683–6.554)	<0.001	0.956 (0.047–19.598)	0.977
Pathologic stage	466				
Stage I & Stage II	267	Reference		Reference	
Stage III & Stage IV	199	2.947 (1.942–4.471)	<0.001	1,085,453,553.6095 (0.000–Inf)	0.981
PAK6	477				
Low	238	Reference		Reference	
High	239	1.803 (1.211–2.682)	0.004	2.651 (0.251–28.041)	0.418
Primary therapy outcome	250				
PD&SD	29	Reference		Reference	
PR&CR	221	0.094 (0.049–0.182)	<0.001	0.020 (0.002–0.168)	<0.001
Gender	477				
Female	226	Reference			
Male	251	1.101 (0.746–1.625)	0.627		
Age	477				
≤65	194	Reference		Reference	
>65	283	1.610 (1.052–2.463)	0.028	2.372 (0.224–25.170)	0.473
Histological type	472				
Adenocarcinoma	402	Reference			
Mucinous adenocarcinoma	70	1.269 (0.753–2.139)	0.371		
Residual tumor	373				
R0	345	Reference		Reference	
R1&R2	28	4.364 (2.401–7.930)	<0.001	1.625 (0.081–32.707)	0.751
Perineural invasion	181				
No	135	Reference		Reference	
Yes	46	1.940 (0.982–3.832)	0.056	1.147 (0.055–23.782)	0.93
Lymphatic invasion	433				
No	265	Reference		Reference	
Yes	168	2.450 (1.614–3.720)	<0.001	17.373 (1.959–154.051)	0.01

* Note: Results for some variables (e.g., Pathologic T stage, Pathologic stage) should be interpreted with extreme caution due to extremely wide confidence intervals from model instability.

**Table 2:** The correlation of clinicopathological characteristics and PAK6 expression in CRC.

Characteristic	Levels	PAK6 Expression		*p*
		Low (*n* = 24)	High (*n* = 25)	
Gender	Male	14 (28.6%)	13 (26.5%)	0.656
	Female	10 (20.4%)	12 (24.5%)	
Age	<60	8 (16.3%)	13 (26.5%)	0.187
	≥60	16 (32.7%)	12 (24.5%)	
Differentiation	Well	0 (0.0%)	5 (10.2%)	0.021 *
	Moderate	24 (49.0%)	20 (40.8%)	
Tumor size	<4 cm	12 (24.5%)	16 (32.7%)	0.322
	≥4 cm	12 (24.5%)	9 (18.4%)	
LVI	No	15 (30.6%)	13 (26.5%)	0.458
	Yes	9 (18.4%)	12 (24.5%)	
PNI	No	19 (38.8%)	17 (34.7%)	0.376
	Yes	5 (10.2%)	8 (16.3%)	
T stage	T1 + T2	4 (8.2%)	4 (8.2%)	0.95
	T3 + T4	20 (40.8%)	21 (42.9%)	
N stage	Negative	15 (30.6%)	14 (28.6%)	0.644
	Positive	9 (18.4%)	11 (22.4%)	
M stage	Negative	20 (40.8%)	19 (38.8%)	0.524
	Positive	4 (8.2%)	6 (12.2%)	
Stage	Early (I + II)	12 (24.5%)	12 (24.5%)	0.889
	Late (III + IV)	12 (24.5%)	13 (26.5%)	

* Statistically significant.

**Table 3:** Correlation between the infiltration level of immune cells and the expression of PAK6 in CRC.

Immune Cell Type	Coefficient of Correlation	*p* Value adj.*
NK CD56bright cells	0.288948	9.9 × 10^−9^
NK CD56dim cells	0.277894	2.6235 × 10^−9^
Th2 cells	0.270829	4.89 × 10^−9^
aDC	0.220401	0.00000243
NK cells	0.207003	0.000008658
Cytotoxic cells	0.177571	0.0001188
pDC	−0.177497	0.0001188
Tcm	−0.157958	0.0005625
TFH	0.13176	0.0038

* *p* values are FDR-adjusted.

## Data Availability

All original data supporting the findings of this study are available from the corresponding author upon reasonable request.
